# Atomic Force Microscopy and High-Resolution Spectrophotometry for Study of Anoxemia and Normoxemia in Model Experiment In Vitro

**DOI:** 10.3390/ijms241311043

**Published:** 2023-07-03

**Authors:** Elena Kozlova, Ekaterina Sherstyukova, Viktoria Sergunova, Andrey Grechko, Artem Kuzovlev, Snezhanna Lyapunova, Vladimir Inozemtsev, Aleksandr Kozlov, Aleksandr Chernysh

**Affiliations:** 1 Laboratory of Biophysics of Cell Membranes under Critical State, Federal Research and Clinical Center of Intensive Care Medicine and Rehabilitology, V.A. Negovsky Research Institute of General Reanimatology, 107031 Moscow, Russia; waterlake@mail.ru (E.K.); kmanchenko@yandex.ru (E.S.); vika_23s82@mail.ru (V.S.); va.inozemcev@physics.msu.ru (V.I.); amchernysh@mail.ru (A.C.); 2 Department of Medical and Biological Physics, Sechenov First Moscow State Medical University, 119991 Moscow, Russia; fillnoise@mail.ru; 3 Faculty of Physics, Federal State Budget Educational Institution of Higher Education M.V. Lomonosov Moscow State University, 119234 Moscow, Russia; 4 Administration, Federal Research and Clinical Center of Intensive Care Medicine and Rehabilitology, 107031 Moscow, Russia; avgrechko@fnkcrr.ru (A.G.); artem_kuzovlev@fnkcrr.ru (A.K.)

**Keywords:** RBCs, hypoxemia, anoxemia, atomic force microscopy, spectrophotometry, hemoglobin derivatives, hemolysis level, optimal hypoxemia level

## Abstract

The oxygen content in the blood may decrease under the influence of various physicochemical factors and different diseases. The state of hypoxemia is especially dangerous for critically ill patients. In this paper, we describe and analyze the changes in the characteristics of red blood cells (RBCs) with decreasing levels of oxygen in the RBC suspension from normoxemia to hypoxemia/anoxemia in an in vitro model experiment. The RBCs were stored in hypoxemia/anoxemia and normoxemia conditions in closed and open tubes correspondingly. For the quantitative study of RBC parameter changes, we used atomic force microscopy, digital spectrophotometry, and nonlinear curve fitting of the optical spectra. In both closed and open tubes, at the end of the storage period by day 29, only 2% of discocytes remained, and mainly irreversible types, such as microspherocytes and ghosts, were observed. RBC hemolysis occurred at a level of 25–30%. Addition of the storage solution, depending on the concentration, changed the influence of hypoxemia on RBCs. The reversibility of the change in hemoglobin derivatives was checked. Based on the experimental data and model approach, we assume that there is an optimal level of hypoxemia at which the imbalance between the oxidative and antioxidant systems, the rate of formation of reactive oxygen species, and, accordingly, the disturbances in RBCs, will be minimal.

## 1. Introduction

The structure and function of red blood cells (RBCs) in the blood are crucial for gas exchange between blood and tissues, especially for critical care patients [1,2,3]. The oxygen content plays an important role in these processes. Critical care medicine deals with all possible oxygen conditions: anoxemia, hypoxemia, normoxemia, and hyperoxemia. Critical illnesses such as major trauma, pneumonia, sepsis, or heart failure can cause dangerously low blood oxygen levels (i.e., hypoxemia), which can cause tissue hypoxia, damage, and death [3,4,5]. In some cases, these terms are still used interchangeably [6]. An extreme case of hypoxemia under an abnormal reduction in the oxygen content of the blood, namely anoxemia, is also possible [7]. To avoid hypoxemia/anoxemia of patients in a critical state, supplemental oxygen is used, leading to hyperoxemia in some cases. However, the idea that both hypoxemia and hyperoxemia may result in complications is accepted [3,4,8,9]. At the same time, the question is raised about the optimal oxygenation goal in various diseases, in particular, acute lung injury, sepsis, stroke, myocardial infarction, etc. [3,4].

The study of structural and functional changes that occur directly in RBCs during hypoxemia is of particular interest. RBCs become hypoxemic for a variety of reasons. One of the main causes of hypoxemia is inadequate blood or oxygen supply to the lungs. Hypoxemia can result from impaired respiratory function, such as that which occurs in patients on artificial lung ventilation [10,11]. Thus, RBCs from COVID-19 patients are in partial hypoxemia, specifically due to reduced gas exchange in the lungs, with the development of tissue hypoxia [12,13,14,15,16]. Hypoxemia and hypoxia can also develop in newborns due to abnormalities during pregnancy [17,18,19,20]. Perinatal hypoxia can affect RBC morphology and membrane nanostructure [21]. Disturbances in blood oxygen delivery may occur at high altitude in the mountains [22,23,24]. Hypoxemia and its consequences should also be considered when treating patients using gases such as helium, xenon, and CO_2_ [25,26,27]. Intermittent hypoxemia is a special situation [28]. The morphology of RBCs, specifically the stiffness and nanostructure of their membranes, can be altered in blood diseases, resulting in impaired cellular deformability [29,30,31,32]. Abnormal content of hemoglobin derivatives in the blood, i.e., increased levels of methemoglobin, deoxyhemoglobin, and carboxyhemoglobin, resulting in a decreased concentration of oxyhemoglobin, may also be observed [33,34,35,36].

The study of RBCs in the hypoxemia state is also related to the issue of long-term storage of packed donor RBCs in vitro. Packed RBCs are stored in special sealed bags with reduced oxygen levels [37,38,39]. During storage, storage lesions and morphologic changes may occur and affect RBCs [40,41,42]. The oxygen permeability of storage bags leads to increasing of RBC membrane disruption and, as the result, to complications during blood transfusion. Recently, new technologies have been developed based on the use of inert gases to remove oxygen from solution for the storage of packed RBCs under hypoxic conditions. The anaerobic storage of RBCs provides a new opportunity to further investigate the mechanisms underlying metabolic storage lesions in order to improve the quality of stored RBCs for transfusion [43,44,45].

Scientific data on the effect of hypoxemia on RBC properties are rather contradictory [22]. On the one hand, hypoxemia causes impaired RBC morphology [46,47]; a decrease in cell deformability [46,48,49,50,51]; damage to membrane proteins and membrane protein cross-linking [22]; damage to the cytoskeleton [47,49]; changes in the lipid composition and extensive peroxidation of membrane lipids [50,52]; an increase in permeability to various ions, ultimately leading to hemolysis [22]; an increase in the level of intracellular 2,3-DPG [53]; the activation of inflammation [54]; deoxyhemoglobin accumulation [55]; methemoglobin formation [56]; and the impaired ability of RBCs to respond to oxidative stress [57,58]. On the other hand, studies have reported that hypoxemia can reduce storage lesions [59,60,61], improve the quality of RBCs in vitro [59], increase the survival of RBCs after transfusion [59], improve the energy and redox metabolism of stored RBCs [62], and have a positive effect on the bulk mechanical properties of stored RBCs [63]. Consequently, hypoxemia may increase the shelf life of RBCs in vitro storage [43,44]. Anaerobic storage could therefore potentially improve the safety and efficacy of blood [63,64].

Oxygen in body organs and tissues, including blood, is the main regulator of redox processes. An increase of oxygen levels in blood leads to higher reactive oxygen species (ROS) production [4]. In the circulation, RBCs are continuously exposed to both endogenous and exogenous ROS, which can damage RBCs and impair their function. To minimize the effects of these ROS and the resulting oxidative stress, RBCs possess an extensive antioxidant system [49,65]. This defense system can prevent oxidative cell damage, resulting in an adequate and balanced redox status. Studies have shown that hypoxemia limits the antioxidant capacity of RBCs [22,66,67]. Under these conditions, redox imbalance increases compared to normoxemia [58]. If there is an imbalance between the production of ROS in biological systems and their ability to defend themselves with a complex antioxidant system, then oxidative stress occurs, leading to structural disorders in cells [68]. The underlying mechanisms of hypoxemia-induced injury and understanding of the response of RBCs to deoxygenation and hypoxemia are still discussed in a number of studies [22].

In this paper, we describe and analyze the changes in RBC characteristics as the oxygen level in the RBC suspension changes from normoxemia to hypoxemia/anoxemia in an in vitro model experiment. These results may help to better understand the role of hypoxemia in the structure and function of RBCs.

## 2. Results

**Figure 1:**
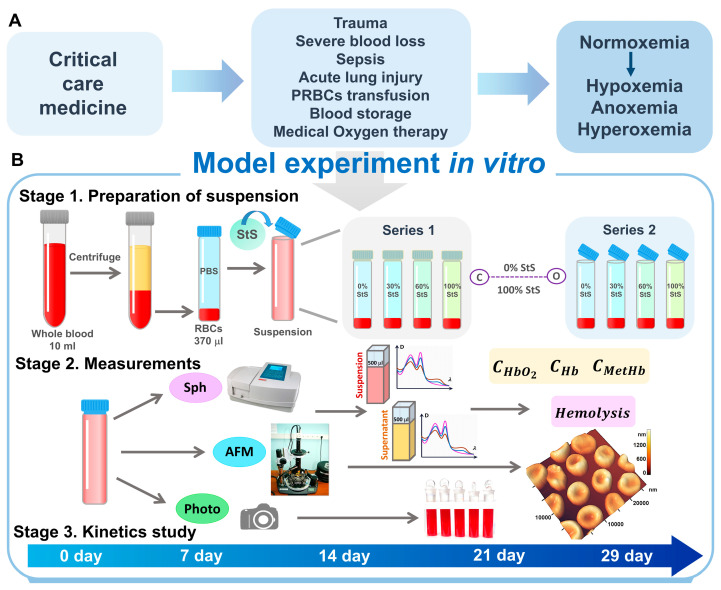
(**A**) Critical care medicine and changes in blood oxygen levels. (**B**) The design of the study. Stage 1: preparation of RBC suspensions with different concentration of storage solutions (StS), two series in closed and open tubes. Stage 2: investigation methods: spectrophotometry (Sph), atomic force microscopy (AFM), and photography. Stage 3: Kinetics of study. The measurements were performed on the specified days.

Critical care medicine deals with various significant abnormalities in the body in which the oxygen content in the blood changes: from normoxemia to hypoxemia/anoxemia, as well as hyperoxemia. In addition, methods of treating critically ill patients are accompanied by a change in the oxygen content in the blood (Figure 1A).

We performed model experiments in vitro according to the stages, indicated in the scheme shown in Figure 1B. In our study, we prepared two types of samples, namely closed and open samples. For closed samples, we used glass tubes with lapped lids. This suspension storage simulated hypoxemic conditions. There was almost no flow of oxygen into the suspension. Conversely, open tubes created conditions for oxygen to flow into the suspension, thus modeling normoxemia. In this model experiment in vitro, RBCs were stored in conditions significantly different from real ones in the body.

### 2.1. Comparison of Changes in RBC Morphology in Open (Normoxemia) and Closed (Hypoxemia/Anoxemia) Glass Tubes

**Figure 2:**
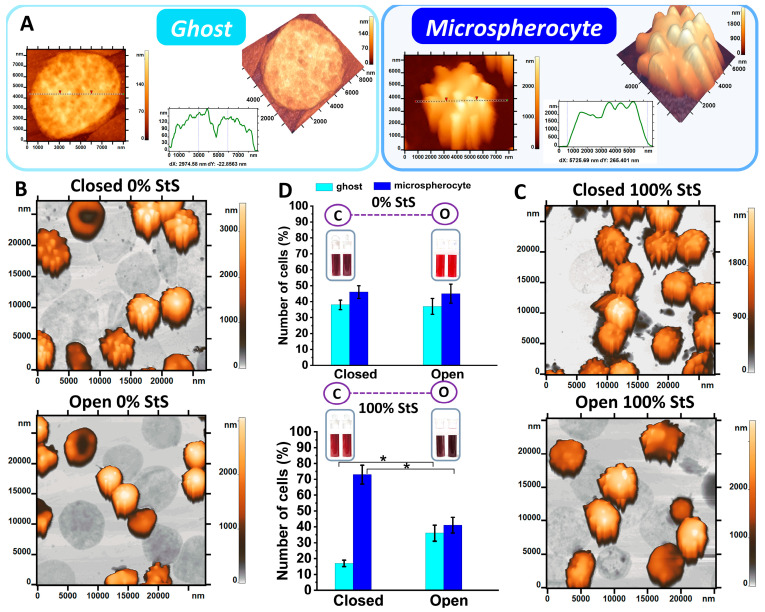
Comparison of the number of common cell shapes (ghosts and microspherocytes) on day 29 in anoxemia and normoxemia. AFM images of RBCs. (**A**) AFM 2D and 3D images of ghosts and microspherocytes and their profiles. (**B**) AFM 2D images of cells in the smear for closed and open tubes at 0% StS on day 29. Scan area of 25 × 25 μm^2^. (**C**) AFM 2D images of cells in the smear for closed and open tubes at 100% StS on day 29. Scan area of 25 × 25 μm^2^. (**D**) Plots of ghost and microspherocyte percentages in the smear for closed and open tubes at 0% and 100% StS on day 29. * *p* < 0.05 (Mann–Whitney test).

By day 29 of storage, the cell morphology changed significantly. Most of the RBCs transformed into ghosts and microspherocytes (Figure 2A). Figure 2 shows a comparison of the percentages of microspherocytes and ghosts on day 29 of storage under anoxemia and normoxemia. The AFM 2D and 3D images and their profiles are shown. Microspherocytes were characterized by a small diameter of about 6 μm and the presence of numerous small regular spike-like projections (Figure 2A). The appearance of ghosts is associated with hemolysis, i.e., destruction of the cell membrane and release of hemoglobin from the cell. Inside the ghosts, there is almost no hemoglobin. The diameter of the RBC ghost was approximately 8 μm, and the thickness was 40–100 nm (Figure 2A).

AFM scan areas 25 × 25 μm^2^ on day 29 of storage are shown in Figure 2B,C for comparison. The histograms show the percentages of cells (%) in open and closed tubes on day 29 at 0% and 100% of storage solution (StS) (Figure 2D). Studies have shown that at 0% StS, the percentages of microspherocytes and ghosts were similar for closed and open tubes (*p* > 0.05) (Figure 2B–D). The results showed that long-term storage of the cells in tubes in a solution without nutrition under the conditions of hypoxemia/anoxemia and normoxemia led to the same result in changing the RBC morphology.

In opened tubes, the storage solution did not influence on this ratio. When 100% StS was added, the percentages of ghosts and microspherocytes for closed and open tubes differed significantly at *p* < 0.05 (Figure 2D). The proportion of ghosts decreased 2.1-fold in closed tubes compared to open tubes. In contrast, the proportion of microspherocytes in the closed tubes was 1.8-fold higher than in the open tubes.

Storage in 100% StS in closed tubes led to changes in the ratio of the RBC type content in comparison with the results in 0% StS. The number of ghosts and microspherocytes in solutions 100% StS and 0% StS was al-most equal in open tubes.

**Figure 3:**
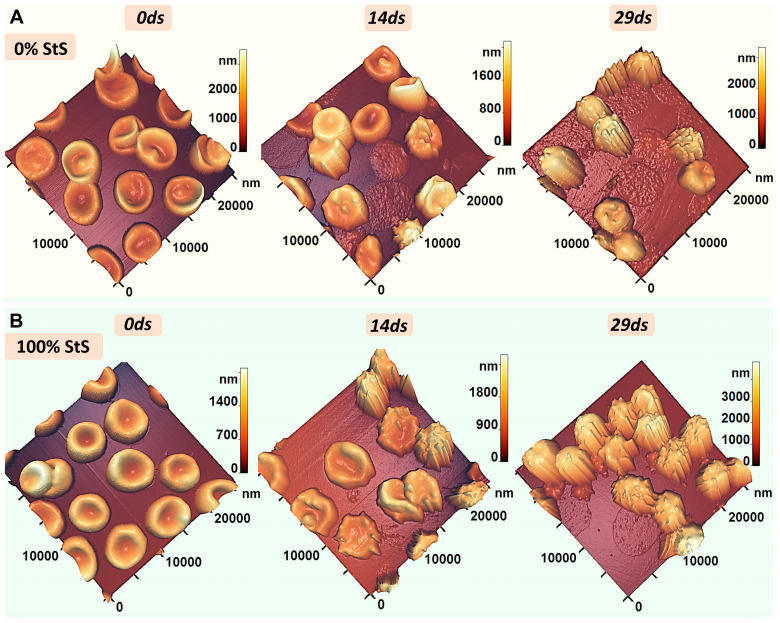
Changes in RBC morphology during storage in closed tubes (hypoxemia/anoxemia). (**A**) AFM 3D images of cells at 0% StS on days 0, 14, and 29. (**B**) AFM 3D images of cells at 100% StS on days 0, 14, and 29.

Under hypoxemia/anoxemia and normoxemia conditions, the RBC morphology changed during storage time. The AFM 3D images of typical 25 × 25 μm^2^ fragments of RBC monolayer smears are shown for 0, 14, and 29 days of storage correspondingly for 0% StS (Figure 3A) and 100% StS (Figure 3B).

**Figure 4:**
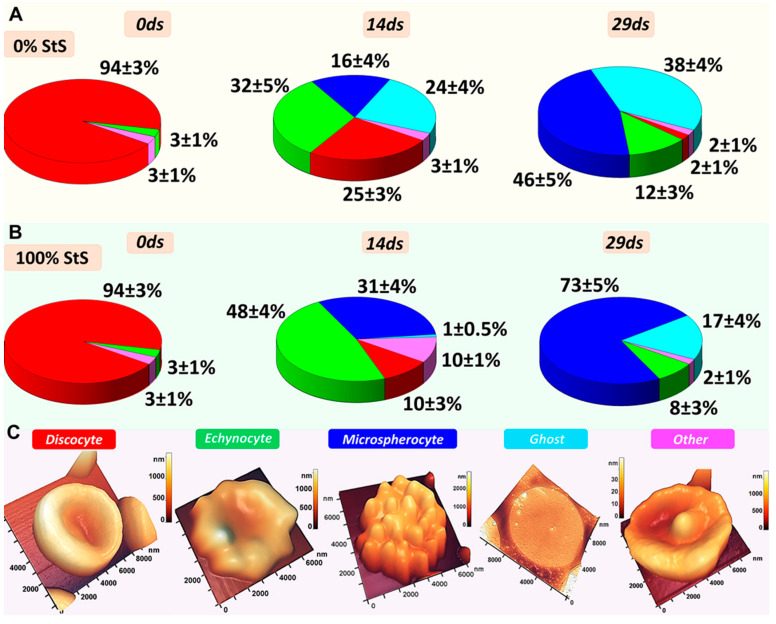
The diagrams of changes in RBC morphology during storage in closed tubes (hypoxemia/anoxemia). (**A**) The diagrams show the percentage (%) of different cell shapes at 0% StS on days 0, 14, and 29. (**B**) The diagrams show the percentage (%) of different cell shapes at 100% StS on days 0, 14, and 29. The color corresponds to the typical cell shapes. (**C**) Typical cell shapes.

Corresponding plots of the percentage of cells of different shapes are presented in Figure 4. Initially, discocytes predominated in all samples (Figure 3 and Figure 4). The number of discocytes was 94 ± 3% (Figure 4). With storage in closed and open tubes, the discocytes changed shape and a polymorphism was observed with the appearance of echinocytes, microspherocytes, ghosts, and others, as shown in Figure 3 and Figure 4.

Thus, in closed tubes (hypoxemia) on day 14 of storage, some of the discocytes transformed into echinocytes and microspherocytes: in 0% StS, 32 ± 5% echinocytes and 16 ± 4% microspherocytes were found, whereas in 100% StS, 48 ± 4% echinocytes and 31 ± 4% microspherocytes were found (Figure 3 and Figure 4). In 0% StS, however, 24 ± 4% of the cells were hemolyzed and turned into ghosts. On day 29 of storage, the smears showed mostly microspherocytes and ghosts. In 0% StS, there were 46 ± 5% microspherocytes and 38 ± 4% ghosts. At 100% StS, the pattern changed, the percentage of microspherocytes increased to 73 ± 5% and the percentage of ghosts decreased to 17 ± 4% (Figure 3 and Figure 4).

It should be noted that similar trends in RBC shape change were observed in open tubes (normoxemia). Initially, most were discocytes, which changed to microspherocytes and ghosts by the end of the storage period. Changes in RBC shape are important to consider when assessing the deformability of cells and the rheological properties of blood in general [69].

### 2.2. Quantitative Estimation of Hypoxemia Level during Storage Time: Quantification of Hemoglobin Derivatives by Nonlinear Curve Fitting of Experimental Optical Spectra

We found that significant changes in RBCs morphology occurred during their long-term storage in opened and closed tubes. It was necessary to understand the hypoxemia level during RBC storage, which was determined by the content of hemoglobin derivatives.

We found that when closed and open samples were stored in different concentrations of StS, there was a change in the optical spectra. It is known that there are characteristic peaks for hemoglobin derivatives in the region of 500–700 nm. The peaks for oxyhemoglobin (${HbO}_{2}$) are located at 542 nm and 577 nm, the peak for deoxyhemoglobin (Hb) are located at 555 nm, the peak for methemoglobin (MetHb) is located at 630 nm [33].

**Figure 5:**
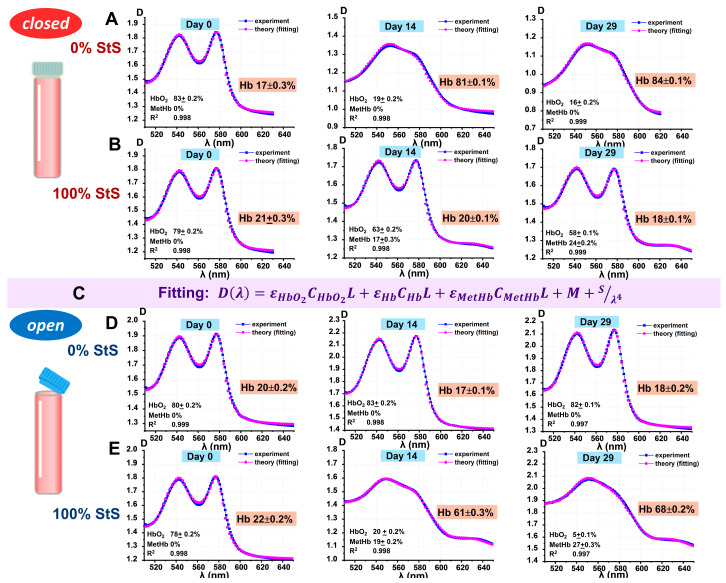
Nonlinear curve fitting of experimental absorption spectra of suspensions for the calculation of hemoglobin derivative levels (examples for closed and open samples on days 0, 14, and 29 of storage). (**A**) Results of calculations for closed samples at 0% StS concentration. (**B**) Results of calculations for closed samples at 100% StS concentration. (**C**) Nonlinear curve fitting equation. (**D**) Results of calculations for open samples at 0% StS concentration. (**E**) Results of calculations for open samples at 100% StS concentration. The levels of the hemoglobin derivatives are shown on each graph as the value ± SE. In addition, the R-squared parameter is shown. Blue dots represent experimental data, pink dots show the fitted curve.

The amplitude of the peaks at 542 nm and 577 nm, which are characteristic of the oxyhemoglobin spectrum, changed. To quantify the levels of hemoglobin derivatives in the tubes, we used nonlinear curve fitting of optical spectra. As an example, Figure 5 shows the results of curve fitting $D_{l}{\left(\lambda_{l}\right)}_{theor}$ for the measured spectra $D_{l}{\left(\lambda_{l}\right)}_{exper}$, which corresponds to the quantitative assessment of hemoglobin derivatives content for closed and open samples at 0% StS and 100% StS concentration on days 0, 14, and 29 of storage.

The theoretical equation is shown in the Materials and Methods and in Figure 5C. The resulting fitting concentrations of hemoglobin derivatives are shown on each graph in Figure 5. The results of the fitting are given as the value ± SE.

### 2.3. Comparison of the Changes in the Levels of Hemoglobin Derivatives in Open and Closed Tubes

**Figure 6:**
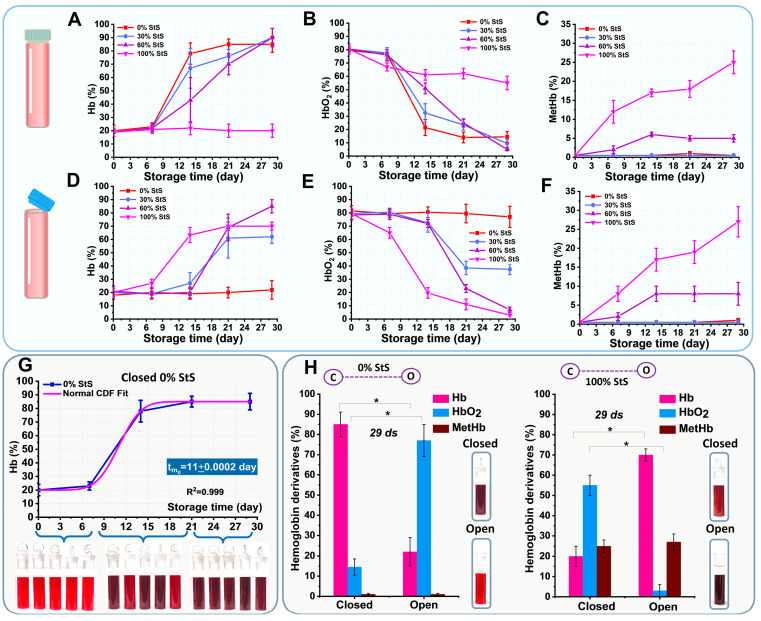
Changes in the percentage of hemoglobin derivatives Hb, ${HbO}_{2},$ and MetHb over time for closed and open samples at different StS. (**A**) Changes in Hb level (%) as a function of storage time in closed tubes. (**B**) Changes in ${HbO}_{2}$ level (%) as a function of storage time (days) in closed tubes. (**C**) Changes in MetHb level (%) as a function of storage time (days) in closed tubes. (**D**) Changes in Hb level (%) as a function of storage time (days) in open tubes. (**E**) Changes in ${HbO}_{2}$ level (%) as a function of storage time (days) in open tubes. (**F**) Changes in MetHb level (%) as a function of storage time (days) in open tubes. (**G**) Results of normal CDF fitting of deoxyhemoglobin levels during storage for 0% StS and photographs of tube colors for three time intervals (indicated by curly brackets). (**H**) Comparison of percentage of hemoglobin derivatives and color of suspension for 0% StS and 100% StS in closed and open tubes on day 29 of storage. Photos of the tubes on this day are shown on the right. Data for each hemoglobin derivatives Hb, ${HbO}_{2}$, and MetHb in closed tubes were compared with corresponding data for open tubes by Mann–Whitney test, * *p* < 0.05.

Quantitative assessment of hemoglobin derivative levels by nonlinear curve fitting allows the study of changes in the conversion of hemoglobin derivatives. Figure 6A–F show how the percentages of the three hemoglobin derivatives ${HbO}_{2}$, Hb, and MetHb changed during storage in all closed and open tubes.

At the beginning of storage, ${HbO}_{2}$ predominated in all samples (80%), and Hb did not exceed 20%. This state corresponds to normoxemia in an in vitro experiment. Then, after 7 days of storage in closed tubes, there was a sharp increase in Hb in 0%, 30%, and 60% StS (Figure 6A). Correspondingly, the ${HbO}_{2}$ level decreased due to developing hypoxemia conditions (Figure 6B). At the same time, Hb remained at the same level for the StS concentration of 100%. From day 7 of storage, MetHb appeared at 60% and 100% StS. On day 29 of storage, the MetHb content was 5 ± 1% in 60% StS and 25 ± 3% in 100% StS (Figure 6C).

In open tubes, Hb increased in 30%, 60%, and 100% StS after 7 days of storage. Thus, on day 29 of storage, the level of Hb reached 62 ± 5% in 30% StS, 85 ± 5% in 60% StS, and 70 ± 3% in 100% StS. This state corresponds to anoxemia. In 0% StS, it remained at the level of 20%. The changes in MetHb were the same as in the closed samples (Figure 6F).

For each concentration, a nonlinear curve fitting was performed using Normal CDF Fit to estimate the average time ($t_{m}$) to reach 50% Hb in the samples. Figure 6G shows an example of nonlinear curve fitting (Normal CDF Fit) for 0% StS in closed tubes with a pink line. Experimental data are shown as mean ± SD with blue dots. For the 0% StS concentration, the mean time was $t_{m_{0}}$ = 11.0 ± 0.5 days (Figure 6G). Keeping RBCs in the storage solution resulted in a longer maintenance of the deoxyhemoglobin level of less than 50%. Thus, for the 30% StS concentration, $t_{m_{30}}$ = 15.2 ± 2.8 days, while for the 60% StS concentration, $t_{m_{60}}$ = 17.7 ± 0.5 days. Interestingly, for the 100% StS concentration, the level of deoxyhemoglobin did not change from the initial level. Thus, the addition of StS decreased the rate of deoxyhemoglobin formation from 0.09 day^−1^ (0% StS) to 0.06 day^−1^ (60% StS).

Photographs of the tubes were taken each day. As shown below in Figure 6G, for 0% StS in the closed tubes, the color of the suspensions changed. During the first week of storage, the color of the suspension was red, corresponding to the high oxyhemoglobin content. During the next two weeks, the suspension in the closed tubes became darker. At the end of storage, the suspension of 0% StS became purple-blue. This change in the color of the suspension indicated the formation of large amounts of deoxyhemoglobin in the anoxemia state.

By day 25, the deoxyhemoglobin (Hb) content had reached saturation in both open (about 20 ± 3%, Figure 6D, red line) and closed tubes (about 85 ± 6%, Figure 6A, red line). We noticed an interesting effect on day 29 of storage, shown in Figure 6H. At 0% StS, Hb formation in the closed (anoxemia) and open (normoxemia) tubes differed with *p* < 0.05. In the closed samples, as Hb predominated (85 ± 6%) and the color of the suspension was purple-blue (Figure 6H). In the open samples, however, the amount of Hb was the same as at the beginning and was 20 ± 3%, while the color of the suspension was red. No additional MetHb was produced in either closed or open tubes. Thus, the increase of deoxyhemoglobin indicated a decreasing concentration of oxygen in the tubes.

When 100% StS was added, the situation was reversed. On day 29 of storage, Hb levels in the closed and open tubes also showed significant differences at *p* < 0.05. However, the level of Hb in the closed tubes was 20 ± 5%, while in the open tubes, it was 70 ± 3%. Interestingly, the addition of 100% StS was accompanied by the production of MetHb, the percentage of which reached 25 ± 3% in closed and 27 ± 4% in open samples (the difference was not significant). The color of the suspensions was red-brown in closed samples and purple-blue-brown in open samples.

### 2.4. Probability of Hemoglobin Derivative Conversion during a Switch from Hypoxemia/Anoxemia to Normoxemia

**Figure 7:**
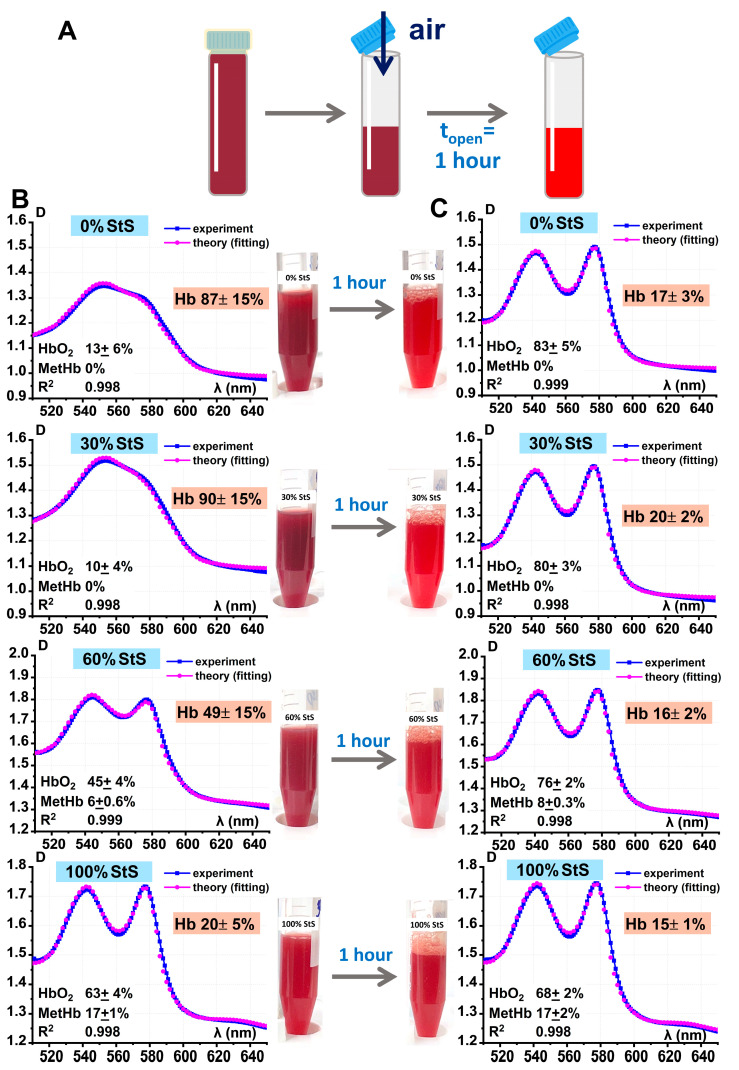
Shift from hypoxemia/anoxemia (closed tubes) to normoxemia (open tubes). Effect on the conversion of hemoglobin derivatives. (**A**) Schematic representation of the shift from hypoxemia/anoxemia to normoxemia. (**B**,**C**) Examples of fitting results of experimental data for 0% StS, 30% StS, 60% StS, and 100% StS samples at baseline (**B**) and after 1 h (**C**), photos of tubes are shown. The levels of hemoglobin derivatives were calculated by the nonlinear curve fitting method and are shown on each graph as value ± SE.

In a direct in vitro biophysical experiment, hypoxemia was found to affect hemoglobin derivative concentrations. What happens when the test tubes are opened, and the oxygen concentration increases again? We experimentally modeled the switch from hypoxemia/anoxemia to normoxemia and presented it in Figure 7A.

Optical absorption spectra of the suspension in closed tubes were measured for hypoxemia/anoxemia for each concentration of StS, t = 0. Hemoglobin derivative concentrations were calculated (Figure 7B). The tubes were then left open for 1 h to switch from hypoxemia/anoxemia to normoxemia (Figure 7A,B). After 1 h, the optical spectra changed, and the hemoglobin derivative concentrations changed accordingly (Figure 7C).

Figure 7B,C show characteristic spectra for each concentration of StS, their calculation, and photos of the suspensions at t = 0 and after 1 h. For the 0% StS concentration, the percentage of Hb decreased from 78 ± 4% to 19 ± 3% after 1 h; for 30% StS, from 67 ± 15% to 21 ± 3%; and for 60% StS, from 43 ± 17% to 19 ± 4%. This meant that there was a recovery of the hemoglobin derivatives, i.e., the process was reversible. In addition, the color of the suspension changed to red, which also indicated the recovery of the hemoglobin derivatives.

Meanwhile, at 100% StS, the percentage of deoxyhemoglobin reduced by a few percent and methemoglobin did not change despite the fact that the oxygen concentration increased to reach normoxemia.

### 2.5. Comparison of Hemolysis Levels during Storage of RBCs in Open and Closed Tubes

During storage of the samples, we observed a change in the color of the supernatant. This was a qualitative indication of the development of hemolysis.

In our study, we quantified the degree of hemolysis *K* (%) based on the optical spectra of the supernatant and suspension using Equation (5) in Materials and Methods.

**Figure 8:**
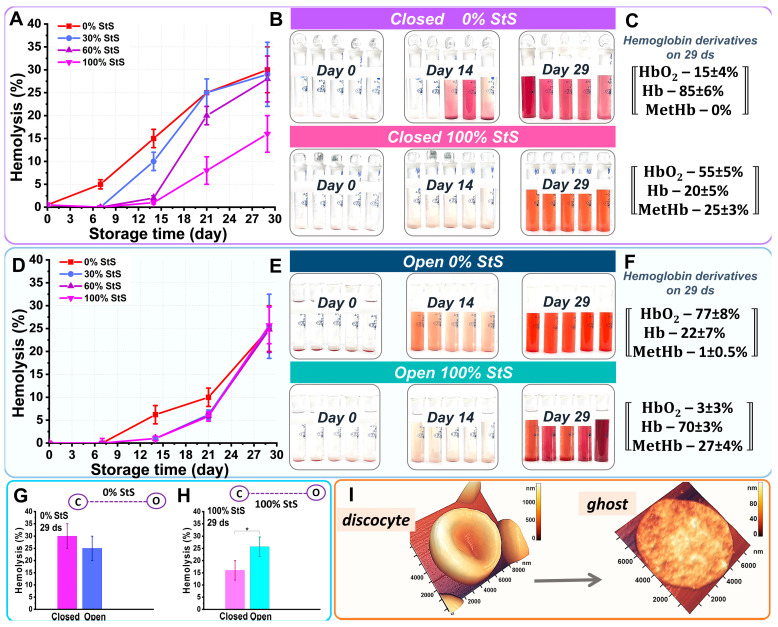
Degree of hemolysis in open and closed tubes as a function of storage time (days). (**A**) Change in hemolysis percentage in samples with 0% StS, 30% StS, 60% StS, and 100% StS in closed tubes. (**B**) Photograph of supernatant in closed tubes at 0% StS and 100% StS concentrations on days 0, 14, and 29. (**C**) Percentage of hemoglobin derivatives on day 29 in closed tubes at 0% StS and 100% StS. Concentration values are shown as mean ± SD. (**D**) Changes in percentage of hemolysis in 0% StS, 30% StS, 60% StS, and 100% StS samples in open tubes. (**E**) Photographs of supernatant in open tubes at 0% StS and 100% StS on days 0, 14, and 29. (**F**) Percentage of hemoglobin derivatives on day 29 in open tubes at 0% StS and 100% StS. Concentration values are shown as mean ± SD. (**G**) Histogram of percentage of hemolysis on day 29 in closed and open tubes at 0% StS. (**H**) Histogram of percentage of hemolysis on day 29 in closed and open tubes at 100% StS. * *p* < 0.05 (Mann–Whitney test). (**I**) Cell shape conversion during hemolysis.

Figure 8A–C show the evolution of the hemolysis level in the closed samples during storage. For the closed tubes, the hemolysis level at the end of storage was 30 ± 5% for 30% StS, 29 ± 7% for 30% StS, and 28 ± 5% for 60% StS. However, when stored in 100% StS, the hemolysis rate was almost half less, 16 ± 4% (Figure 8A).

Figure 8B shows a comparison of the color of the supernatant on different days of storage for the 0% and 100% StS suspensions. Starting on day 14 of storage in the 0% StS suspension, the color of the supernatant changed dramatically to purple-blue. As the concentration of StS in the suspension increased, the color change of the supernatant began later. For 100% StS, the supernatant began to change color after 20 days of storage and was a light red-brown color.

Figure 8D–F shows how the degree of hemolysis changed in the open samples during storage. For the open tubes, the rate of hemolysis at the end of storage was 25 ± 5% for all samples with different StS (Figure 8D).

Figure 8E shows a comparison of supernatant color at different storage days for the 0% and 100% StS suspensions. Starting on day 14 of storage for 0% StS, the color of the supernatant began to change to pale red, and on day 29 of storage, the supernatant became bright red. In tubes with 30%, 60%, and 100% StS, the color of the supernatant began to change around day 20 of storage. By day 29 of storage, the color of the supernatant was burgundy-purple.

The color change of the supernatant on day 29 was determined by the percentage of hemoglobin derivatives in the suspension (Figure 8C,F).

No significant difference was observed in the percentage of hemolysis in the closed and open tubes for 0% StS (Figure 8G). However, the percentage of hemolysis in the closed and open tubes for StS 100% differed at *p* < 0.05 (Figure 8H).

During cell hemolysis, the original discocytes eventually turned into ghosts (Figure 8I). The ghost percentage data corresponded well with the hemolysis data (Figure 3).

## 3. Discussion

### 3.1. Anoxemia, Hypoxemia, Normoxemia, Hyperoxemia, Redox Balance, and ROS

We showed in model experiments in vitro that irreversible changes occurred in RBCs at the end of storage (day 29) in Solution 0% StS in glass tubes with tightly closed lids, i.e., in hypoxemia/anoxemia conditions. The cell morphology changed significantly. At the end of the storage period (day 29), only 2% of discocytes remained, and irreversible types, such as microspherocytes and ghosts, appeared (Figure 2, Figure 3 and Figure 4). RBC hemolysis occurred at a value of 25–30% (Figure 8). Remarkably, the same results were obtained in in vitro experiments in glass tubes with completely open lids (0% StS), i.e., under normoxemia (Figure 8D–F). In both cases, almost no methemoglobin appeared at the end of the storage time (Figure 8). The only difference was the level of deoxyhemoglobin, which was about 80–90% in hypoxemia/anoxemia and about 18% in normoxemia, which is quite natural under these experimental conditions.

Interestingly, the addition of the storage solution altered the kinetic parameters depending on its concentration (Figure 2, Figure 3, Figure 4, Figure 6 and Figure 8).

The mechanism of changes in RBCs under hypoxemia/anoxemia and hyperoxemia in comparison with normoxemia is based primarily on the ratio of oxidative/antioxidant system performance under these conditions [68]. An imbalance between oxidant and antioxidant systems leads to oxidative stress, that is, to a sharp increase of ROS [70].

The hypoxemia results in the imbalance between $O_{2}$ supply and requirements, which could induce tissue hypoxia and cell death. On the other hand, the presence of hyperoxemia enhances ROS and oxidative stress, which cause alveolar and cell damage [9].

Under hyperoxemia, the oxidative system is enhanced. This results in redox imbalance. The production of ROS is increased. Coexisting lung inflammation may lower the threshold for oxygen toxicity in patients with acute respiratory distress syndrome or in other acute illnesses in the lung [71].

What happens in hypoxemia/anoxemia? Hypoxemia/anoxemia limits the antioxidant capacity of red blood cells [66,67]. This also results in redox imbalance and, as a consequence, oxidative stress [58,67]. Under hypoxemia, the superoxide production dramatically increases [48,49]. As a result, RBCs are exposed to intense oxidation under reduced oxygen pressure [48], and the rate of Hb autoxidation increases [49]. During anoxic shock, the dramatic increase of ROS was also observed in other biological objects [72].

Anoxemia and hyperoxemia result in imbalances of oxidative/antioxidant system functioning, leading to cell disturbances.

RBCs are susceptible to oxidative damage due to the effects of heme-bound and free iron, high levels of molecular oxygen in hemoglobin, and polyunsaturated fatty acids in membranes [73]. Changes in membrane structure as a result of oxidative stress occur both when RBCs are exposed to oxidizing agents and after incubation under anaerobic conditions [18,71,74]. The superoxide, $H_{2}O_{2}$, hydroxyl radicals, and other ROS generated by redox reactions can damage RBC membrane proteins, lipids, and the cytoskeleton, which are responsible for maintaining RBC shape and deformability. Partially oxygenated hemo-globin may have a higher affinity for band 3 than the normal one [49,74,75]. The interaction of Hb, especially under hypoxic conditions, with band 3 of the RBC membrane is critical for inducing RBC membrane changes [49].

As a result, the concentrations of hemoglobin derivatives and the level of hemolysis will change, and polymorphism will occur (Figure 2, Figure 3, Figure 4, Figure 6 and Figure 8).

### 3.2. Kinetic Model of ROS Change. Redox Imbalance. Hypothesis of Hypoxemia Optimal Level

**Figure 9:**
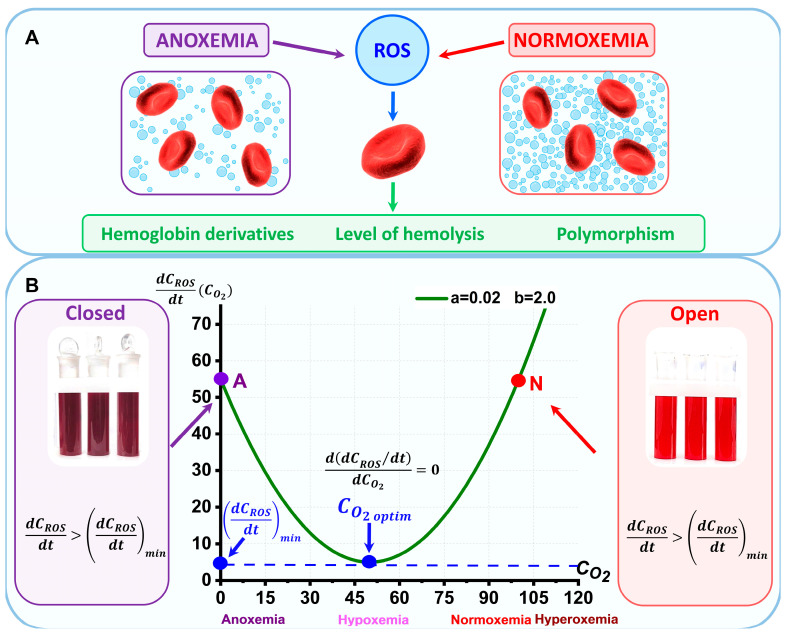
Model representation of the effect of oxygen concentration $C_{O_{2}}$ on the rate of ROS formation. (**A**) Schematic representation of ROS generation during hypoxemia/anoxemia and normoxemia and its effect on RBC parameters. (**B**) Relationship between ROS generation rate and oxygen concentration. Point N corresponds to the oxygen level in normoxemia, point A indicates anoxemia. The blue point indicates the optimal oxygen level at which the rate of ROS generation is minimal (exactly for these indicated parameters: a = 0.02 and b = 2.0). The purple dot indicates anoxemia conditions. The red dot indicates normoxemia conditions. All quantities are given in arbitrary units. The corresponding photographs of the tubes are shown on the left and right.

Experiments showed that, when stored in PBS in open and completely closed tubes, the RBC lesions were almost identical in the conditions of our experiments. Severe hemolysis occurred, and cell morphology was significantly distorted. This indicates that oxidation occurs with approximately the same degree both at normoxemia oxygen concentration ($C_{O_{2\;normoxemia}}$) and at anoxemia oxygen concentration ($C_{O_{2\;anoxemia}}$), (Figure 9). This means that unfavorable conditions for RBCs storage occur both at normal oxygen concentration and in the absence of oxygen. At the same time, it is known that during the long-term storage of packed RBCs in sealed bags with the storage solution, quite different quantitative results are observed. In this study, in the open and closed tubes, hemolysis was about 30% by day 29 of storage. In the sealed storage bags, the degree of hemolysis was below 1% [30]. This indicates that the non-monotonic dependence of the RBCs parameters changes in the dependence of oxygen content during storage. It is important that this hypothesis of the optimal oxygen concentration for the lowest rate of ROS creation and, consequently, small damages in the RBC, is discussed in the model.

An interesting question arises about the comparison of the results of storage by day 29 under conditions of different oxygen content in closed (anoxemia) and open (normoxemia) tubes and in sealed storage bags (moderate hypoxemia). It is under these different conditions that RBCs are in solutions with different amounts of oxygen:

$$C_{O_{2}\;open}>C_{O_{2}\;bag}>C_{O_{2}\;closed}.$$

It can be assumed that these significant differences are primarily due to the different levels of hypoxemia in the storage bags and in the glass tubes.

Based on the revealed patterns, we hypothesize that there is an optimal level of oxygen corresponding with the hypoxemia level, $C_{O_{2}optim}$, at which the damage to RBCs during storage is minimal. In other words, it is the level of hypoxemia, not just the hypoxemia itself, that is important during RBC storage and during their lifetime.

Let us consider a simple mathematical model illustrating the rate of ROS change during oxidative/antioxidant imbalance with changes in oxygen concentration $C_{O_{2}}$, which corresponds to the partial pressure of oxygen in the solution.

This approach allows a qualitative interpretation of the existence of the optimal level of oxygen concentration. Oxidative stress in biological systems can be described as an imbalance between the production of ROS and their ability to protect themselves with an antioxidant system [68].

The work of both oxidant and antioxidant systems depends on the oxygen concentration. With an increase in oxygen concentration, the intensity of the oxidant system increases. With a decrease in oxygen concentration, the intensity of the antioxidant system decreases. As a result, the rate of change in ROS and, accordingly, their number will change with changes in the level of hypoxemia.

Let us make the following assumptions in the model.

(1) Let us consider the functioning of the oxidative system, determined by the oxygen concentration. As a result of the oxidative system, the *ROS* are generated depending on the oxygen concentration $C_{O_{2}}$ at a rate $F_{OS}\left(C_{O_{2}}\right)$.
(2) Let us consider the functioning of the antioxidant system determined by the oxygen concentration. As a result of antioxidant system, the ROS are eliminated depending on the oxygen concentration $C_{O_{2}}$ at a rate $F_{aos}\left(C_{O_{2}}\right)$.
(3) Let $F_{0S}\left(C_{O_{2}}\right)=aC_{O_{2}}^{2}$, where *a* is the oxidant system performance constant. Let $F_{aos}\left(C_{O_{2}}\right)=bC_{O_{2}}$, where *b* is the antioxidant system performance constant.

Then, the balance equation for *ROS* will be:

(1) $$\frac{dC_{ROS}}{dt}=F_{os}\left(C_{O_{2}}\right)-F_{aos}\left(C_{O_{2}}\right)+F_{0}$$

where $F_{0}=const$.

Let us rewrite Equation (1) with assumption and analyze the function:

(2) $$\frac{dC_{ROS}}{dt}=F\left(C_{O_{2}}\right)=aC_{O_{2}}^{2}-bC_{O_{2}}+F_{0}$$

Figure 9B shows function (2) for the values of parameters a = 0.02, b = 2, and $F_{0}=55.$ On the graph, ${\;C}_{O_{2}}$ is the units corresponding to oxygen pressure${\;P}_{O_{2}}$ (mmHg).

The minimum of $F\left(C_{O_{2}}\right)$ will be $C_{O_{2}min}=C_{O_{2}optim}=\frac{b}{2a}$.

At $C_{O_{2}optim}$, the rate of *ROS* generation will be minimal and equal to:

(3) $$({{\frac{dC_{ROS}}{dt})}_{min}=F\left(C_{O_{2}}\right)}_{min}=F_{0}-\frac{b^{2}}{4a}$$

Thus, the rate of ROS production increases with $O_{2}$ excess and with its deficit. This coincides with other studies [3,4,8].

The optimal hypoxemia level $C_{O_{2}optim\;}$and, correspondingly, ${\left(\frac{dC_{ROS}}{dt}\right)}_{min}$, will depend on the intensity of the oxidant system functioning and antioxidant system effectiveness. Storage conditions, such as temperature and the kind and content of the RBC storage solution, will affect these values. This model adequately describes the hypothesis that there is an optimal degree of hypoxemia at which the rate of ROS production is minimal.

There are at least two-target oxygen content problems. The scientifically important and interesting problem is that the oxygen concentration $C_{O_{2}}$ must satisfy the following requirements: it must be $C_{O_{2optim}}$, for which the concentration of ROS should be as low as possible $C_{ROSmin}$, and at the same, $C_{O_{2}}$ must be large enough to supply required oxygen amount to the tissue $C_{O_{2}suppl}$. The difference between these values must be minimal ${{∆}_{min}=C_{O_{2}suppl}-C}_{O_{2}optim}$. The problem of optimal oxygen content is now being discussed in clinical studies [8,9,71]. Optimal oxygenation values when supplemental oxygen is needed will depend on the specific circumstances. In clinics, supplementary oxygen for most patients is necessary if the saturation level is ${SaO}_{2}$ ≤ 94%. Oxygen therapy in patients with acute stroke or acute myocardial infarction starts if ${SaO}_{2}$ ≤ 90–92%, and the threshold in patients who are at risk of hypercapnic respiratory failure falls to 88% [3].

Our research will be useful in studying the problem of optimal oxygenation levels for the development of methods of personalized critical care medicine [76].

## 4. Materials and Methods

### 4.1. Stages of Experiments

The stages of the experiment are shown schematically in Figure 1.

In the first stage, RBC suspension was prepared and then poured into glass tubes. PBS was added (control samples), and storage solution (StS) in different concentrations was added to some tubes. Two sets of tubes were prepared: some tubes were closed with lapped lids wrapped in paraffin (C), and some tubes were opened (O). Figure 1, Figure 2, Figure 3 and Figure 4, Figure 6 and Figure 8 show a schematic comparison of the results for the closed and open tubes with a purple dotted line at 0% StS and 100% StS. Hypoxemia/anoxemia conditions were modeled in the closed tubes, and normoxemia was modeled in the open tubes. The RBCs in the tubes were stored for 29 days. To study the evolution of changes in the RBC parameters on days 0, 7, 14, 21, and 29, the new corresponding tubes were opened, and certain parameters were measured. Thus, in our study, we used different sampling tubes for each time point. In the experiments, we compared cell parameters during hypoxemia/anoxemia in comparison with normoxemia: the levels of hemoglobin derivatives, percentage of hemolysis, and percentage of different cell types. Photographs of the samples were taken daily throughout the experiment.

### 4.2. Preparation of RBC Suspension

The preparation of the suspension is shown schematically in Figure 1B. All experiments were performed in accordance with the guidelines and regulations of the Federal Research and Clinical Center of Intensive Care Medicine and Rehabilitation, V.A. Negovsky Scientific Research Institute of General Reanimatology, Moscow, Russia. All experimental protocols were approved by the institute.

Blood from 6 healthy donors (4 men and 2 women, aged 25–51 years) was used for in vitro experiments studying the changes in RBC suspension during storage. Whole blood (10 mL) was collected in vacutainer EDTA blood collection tubes during routine medical examinations. Informed consent was obtained from each donor. Blood hematocrit was 38–43%.

In the first step of the experiment, 10 mL of whole blood was centrifuged at 2000 rpm for 5 min in a Universal 320 centrifuge (Andreas Hettich GmbH & Co. KG, Tuttlingen, Germany) to separate plasma from RBCs. Then, for all the experiments, we used RBCs without any white blood cells and platelets.

Two sets of tubes were prepared, closed and open. Then, 370 μL of RBCs, PBS pH 7.4 solution (MP Biomedicals LLC, Illkirch-Graffenstaden, France), and storage solution at the appropriate concentration were added to each tube. The storage solution consisted of 63 mL CPD (citrate-phosphate-dextrose) and 100 mL SAGM (saline, adenine, glucose, mannitol) solutions.

There were 4 concentrations in total: the control sample (0% StS) was prepared by adding 12 mL of PBS to RBCs with no added storage solution; 30% StS was prepared by adding 8.4 mL of PBS and 3.6 mL of storage solution to RBCs; 60% StS was prepared by adding 4.8 mL of PBS and 7.2 mL of storage solution to RBCs; 100% StS was prepared by adding 12 mL of storage solution to RBCs. The resulting hematocrit in the RBC suspension was about 0.013.

In each series, 25 tubes were prepared for each StS. Tubes were stirred every day. There were stored at a temperature of +4 °C in refrigerator for blood storage XK-250-1 «POZIS» (POZIS, Zelenodolsk, Russia). Measurements were made on days 0, 7, 14, 21, and 29 at room temperature. Five closed and five open tubes of each concentration were taken on the day of measurement.

### 4.3. Spectrophotometry and Nonlinear Curve Fitting Method for the Estimation of the Hemoglobin Derivative Levels

The absorption spectrum of RBC suspension $D{\left(\lambda \right)}_{exp}$ was measured using a digital spectrophotometer Unico 2800 (United Products& Instruments, Dayton, OH, USA) in 1 nm increments in the wavelength range of 500–700 nm. To measure the spectra, 500 μL of suspension was poured into a quartz cuvette very fast (10 s). The cuvette covered with elastic film PARAFILM M (Pechiney Plastic Packaging, Chicago, IL, USA) was placed in the spectrophotometer.

We analyzed the levels of three hemoglobin derivatives in RBCs: oxyhemoglobin (${HbO}_{2}$), deoxyhemoglobin (Hb), and methemoglobin (MetHb). The nonlinear curve fitting method by Origin Pro 2019 (OriginLab Corporation, Northampton, MA, USA, software 9.8.0.200.) was used to determine hemoglobin derivatives [33]. The theoretical function for approximation $D_{l}{\left(\lambda_{l}\right)}_{theor}$ was calculated using Equation (4):

(4) Dl(λl)theor=ɛHbO2,lCHbO2L+ɛHblCHbL+ɛMetHblCMetHbL+M+Sλ4.

In this equation, several variables are known, such as the molar absorption coefficients at given wavelengths $\lambda_{l}$ (${ɛ}_{HbO_{2},l},\;{ɛ}_{Hb,l},\;{ɛ}_{MetHb,l}$) and the thickness of the layer *L*. The individual absorptivity of the different components were taken from [34]. The thickness of the layer *L* was 1 mm. Since RBCs are studied in PBS, in addition to absorption processes, scattering processes must also be taken into account, which will be different for different wavelengths. The coefficient *M* describes the scattering of light by RBCs when the wavelength is smaller than the RBC diameter d, λ ≪ d. The coefficient *S* corresponds to Rayleigh scattering when the size of the scattering object d’ (roughness of the RBC membrane) is small, λ ≫ d’. Thus, as a result of the approximation according to Equation (1), the unknown model variables were calculated, namely the values of the corresponding hemoglobin derivatives ($C_{HbO_{2}}$, $C_{Hb}$, $C_{MetHb}$) and the scattering coefficients (*M* and *S*), which are given as values ± SE. The fitting curves were constructed on the basis of these calculated values.

To calculate the percentage of hemolysis (*K*), the spectra of the supernatant were measured. The percentage of hemolysis was calculated using the equation:

(5) $$K=\frac{Concentration\;of\;total\;hemoglobin\;in\;the\;supernatant}{Concentration\;of\;total\;hemoglobin\;in\;the\;suspension}\cdot 100\%.$$

### 4.4. Investigation of RBC Morphology by Atomic Force Microscopy

The atomic force microscope NTEGRA Prima (NT-MDT Spectrum Instruments, Moscow, Russia) was used to evaluate RBC morphology. We used NSG01 cantilevers with a gold reflective coating, tip curvature radius of 10 nm, tip height of 14–16 µm, and force constant of 1.45–15.1 N/m (TipsNano, Tallinn, Estonia). Scanning was performed in semi-contact mode. The resonance frequency was 87–230 kHz. The scanning areas ranged from 50 × 50 to 10 × 10 μm^2^. The resolution of image acquisition was 512 and 1024 pixels for each sample. The resulting images were processed using FemtoScan Online software, Version 2.3.239(5.2) (Femtoscan, Moscow, Russia).

To prepare smears, cells were fixed. For this purpose, 50 µL of 1% glutaraldehyde solution (Panreac Quimica S.L.U., Barcelona, Spain) was added to 50 µL of cells. The cells were incubated for 4 min. Then, the samples were washed with distilled water to avoid salts on the smear. A cell monolayer for scanning by atomic force microscope was obtained using a V-Sampler (Vision, Vienna, Austria) by placing a 10 µL specimen on a slide. Dry smears were scanned at room temperature (20 °C).

### 4.5. Statistics

#### 4.5.1. Statistical Analysis

Statistical analysis of the results was performed using OriginPro 2019. Statistical data for samples are given as mean with standard deviation (mean ± SD). The Mann–Whitney nonparametric test was used to test the significance of differences between the means in different groups. Differences were considered significant at *p* < 0.05.

#### 4.5.2. Sample Statistics of RBC Morphology

Three smears of cell monolayer were prepared for each test tube on the corresponding measurement day. Three 50 × 50 μm^2^ scans were obtained for each smear, and 25 × 25 μm^2^ areas and individual cells in the 10 × 10 μm^2^ area were also scanned. In total, about 1050 images were scanned. The statistical analysis of cell shapes was performed.

#### 4.5.3. Sample Statistics of Hemoglobin Derivative Concentrations

Three absorption spectra of the suspension were measured for each test tube on the corresponding measurement day. A total of 1500 optical spectra was analyzed.

## 5. Conclusions

Our study showed that RBCs undergo significant changes under hypoxemia/anoxemia and normoxemia storage in the model experiment in vitro. These include morphological changes such as the appearance of irreversible cell types, namely microspherocytes and ghosts. Changes in the concentration of hemoglobin derivatives also occurred. A high degree of hemolysis was observed. These processes were influenced by the contents of the additive storage solution. In the kinetic model, we hypothesize that there is an optimal level of hypoxemia at which the imbalance between the oxidative and antioxidant systems, the rate of formation of reactive oxygen species, and, accordingly, the disturbances in RBCs, will be minimal. This approach is important for selecting the optimal conditions of donor RBCs storage, the treatment of critically ill patients, developing the strategy of gas treatment, and analyzing the basic mechanisms of RBC functioning in blood.

## Data Availability

The datasets used and analyzed during the current study are available from the corresponding author upon request.
